# Selection and characterization of DNA aptamer against glucagon receptor by cell-SELEX

**DOI:** 10.1038/s41598-017-05840-w

**Published:** 2017-08-03

**Authors:** Guodong Wang, Jun Liu, Ke Chen, Yiling Xu, Bo Liu, Jie Liao, Lei Zhu, Xiaoxiao Hu, Jianglin Li, Ying Pu, Wen Zhong, Ting Fu, Huixia Liu, Weihong Tan

**Affiliations:** 1 Xiangya Hospital, Central South University, Changsha, Hunan 410008 China; 2 Molecular Science and Biomedicine Laboratory, State Key Laboratory for Chemo/Bio-Sensing and Chemometrics, College of Chemistry and Chemical Engineering, College of Biology, and Collaborative Research Center of Molecular Engineering for Theranostics, Hunan University, Changsha, Hunan 410082 China; 3 Anhui Provincial Engineering Research Center for Polysaccharide Drugs, Anhui Province Key Laboratory of Active Biological Macromolecules, School of Pharmacy, Wannan Medical College, Wuhu, 241002 China; 4 Department of Chemistry, Department of Physiology and Functional Genomics, Center for Research at the Bio/Nano Interface, Shands Cancer Center, University of Florida Genetics Institute and McKnight Brain Institute, University of Florida, Gainesville, Florida 32611-7200 United States

## Abstract

Excessive secretion of glucagon, a functional insulin antagonist, significantly contributes to hyperglycemia. Glucagon exerts its physiological functions through activation of the glucagon receptor (GCGR). Inhibition of GCGR activity represents a potential therapeutic approach for reducing excess glucose production in diabetes mellitus. Aptamers are short DNA or RNA oligonucleotides evolved from systematic evolution of ligands by exponential enrichment (SELEX). Here, we have successfully selected a DNA aptamer against GCGR by cell-SELEX, which can specifically bind membrane protein of CHO-GCGR cells with a *K*_*d*_ of 52.7 ± 5.1 nM. Aptamer-mediated pull-down and *gcgr* knockdown assay verified that GCGR was the target of aptamer GR-3. Binding analysis revealed that GR-3 could recognize other cells with different affinity according to the level of GCGR protein expressed in these cells. Hepatic tissue imaging suggested that GR-3 could bind the cell membrane of hepatic tissues. With the advantages of small size, high binding affinity, good stability, lack of immunogenicity, and easy synthesis, aptamer GR-3 against GCGR can be a promising tool with the potential to attenuate hyperglycemia in diabetes mellitus.

## Introduction

Glucagon, a 29-amino acid peptide secreted from pancreatic α cells, is a pivotal counter-regulatory hormone in the regulation of glucose homeostasis^1^. Glucagon stimulates hepatic glucose production and output by promoting glycogenolysis and gluconeogenesis (GNG) in the liver and attenuates the ability of insulin to inhibit these processes in the fasting state^2^. Glucagon exerts its physiological functions through activation of the glucagon receptor (GCGR), which is predominantly localized in the liver^3, 4^. GCGR is a seven trans-membrane G protein-coupled receptor consisting of 485 amino acids. In patients with type 2 diabetes mellitus (T2DM), the secretion of glucagon is increased in both the fasting and postprandial states and contributes to pathogenesis of diabetic hyperglycemia through excessive hepatic glucose production and output^5, 6^. Recent studies revealed that excessive glucagon secretion or action, rather than insulin deficiency, is predominant in the progress of diabetes^7, 8^. Accordingly, inhibition of GCGR activity represents a potential therapeutic approach for reducing excess glucose production in patients with T2DM. For instance, reduction in GCGR expression using antisense oligonucleotides (ASOs) has been shown to lower glycemia and ameliorate metabolic syndrome in *db/db* mice and Zucker diabetic fatty rats^9–11^. Extensive efforts have been undertaken by the pharmaceutical industry to develop potent small molecule glucagon receptor antagonists or antibodies for clinical use^12, 13^. Several glucagon receptor antagonists and antibodies able to improve glucose homeostasis in animal models and humans have been reported^14–16^. However, thus far none has progressed to final marketing approval, mainly due to a poor performance profile, including toxicity or lack of selectivity^17^.

Aptamers are short DNA or RNA oligonucleotides evolved from random oligonucleotide libraries by a process called systematic evolution of ligands by exponential enrichment (SELEX)^18, 19^. They can act as ligands with specific and high binding affinity for a variety of targets, including small molecules, proteins, nucleic acids, viruses, bacteria, cells and tissues^20, 21^. The molecular recognition properties of aptamers are similar to those of antibodies. However, manmade aptamers possess several advantages over naturally occurring antibodies, including economical and reproducible synthesis, easy modification, low toxicity, high stability, lack of immunogenicity, and rapid tissue penetration^22, 23^. In addition to recognition, some aptamers are able to retain their function to regulate biological pathways and interfere with disease development through binding to molecular targets involved in pathogenesis^24^. Based on these advantages, aptamers show high potential for therapeutic applications, such as targeted therapy, detection and diagnostics^25–28^. Macugen, the first aptamer-based drug approved by the U.S. Food and Drug Administration (FDA) in 2004, is now available for treatment of age-related macular degeneration (AMD)^29^. Other aptamers, such as aptamer AS1411, which is specific for nucleolin, are currently undergoing clinical evaluation^30^. This indicates that aptamers can also be used directly as drugs^18^.

For the selection of anti-protein aptamers, SELEX is usually carried out using purified recombinant proteins. Therefore, the precondition of SELEX for anti-protein aptamers is the preparation of sufficient amounts of high-quality, purified protein^31^. However, many pharmacologically relevant cell membrane proteins, such as G protein-coupled receptors, cannot be purified because of their associated complexity and instability^32^. However, SELEX against live cells (cell-SELEX) has enabled the generation of aptamers which, with their flexible conformations, can specifically bind target molecules in their native state on the cell surface without prior knowledge of the molecular signatures of target cells^18, 33^. As such, target molecules do not require purification or anchorage on a solid support by processes that may destroy their native conformations^34^. Additionally, some aptamers selected by cell-SELEX strategies are endowed with inhibitory activity by binding with their cognate cell surface receptor^35, 36^. Owing to these merits, cell-SELEX technology is now used worldwide, and many aptamers against various proteins on different cells have been generated by this method, including epidermal growth factor receptor (EGFR)^37, 38^, epithelial cell adhesion molecule (EpCAM)^39^, platelet-derived growth factor receptor β (PDGFRβ)^36^, insulin receptor^40^, Annexin A2^41^, HER2^42, 43^, and CD133^44^. In this study, a DNA aptamer against GCGR was developed by cell-SELEX, and its physical and biological properties were further investigated.

## Results and Discussion

### Expression of GCGR in transfected cells

To effectively select aptamers specific for GCGR, we sought to express GCGR protein on the surface of CHO-K1 cells in order to maintain its native conformation. CHO-K1 cells were transfected with pcDNA3.1-GCGR, resulting in clones of CHO-GCGR. After transfection, we confirmed the overexpression and location of GCGR by Western blot and confocal microscopy imaging, respectively. As expected, the expression of GCGR protein was significantly increased in CHO-GCGR cells transfected with pcDNA3.1-GCGR. However, the GCGR protein was scarcely detected at all in Mock cells transfected with pcDNA3.1 plasmid (Supplementary Fig. S1A). While red fluorescence was visualized on the cytomembrane of CHO-GCGR, almost no fluorescence was detected in Mock cells (Supplementary Fig. S1B), indicating high GCGR cell-surface expression in the CHO-GCGR cells.

### Selection of DNA aptamer against GCGR-expressing CHO-K1 cells

**Figure 1:**
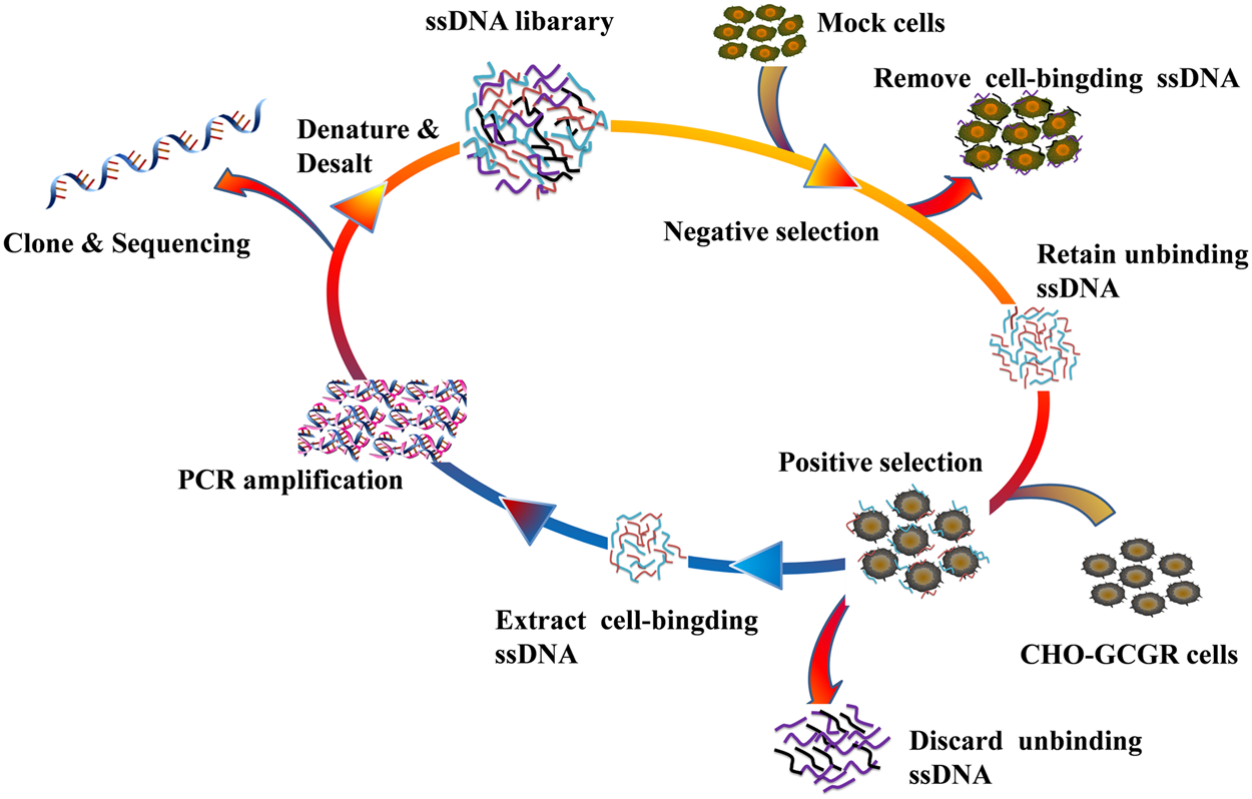
Scheme of cell-SELEX for GCGR-expressing CHO-K1 cells (CHO-GCGR). The ssDNA library was incubated with Mock cells as negative selection to remove the cell-binding ssDNA. The unbound ssDNA was incubated with GCGR-expressing CHO-K1 cells (CHO-GCGR) for positive selection. After washing, the bound DNA was eluted and amplified by PCR for next-round selection. The evolved ssDNA pool was sequenced to identify the aptamer candidates in the last round of selection.

**Figure 2:**
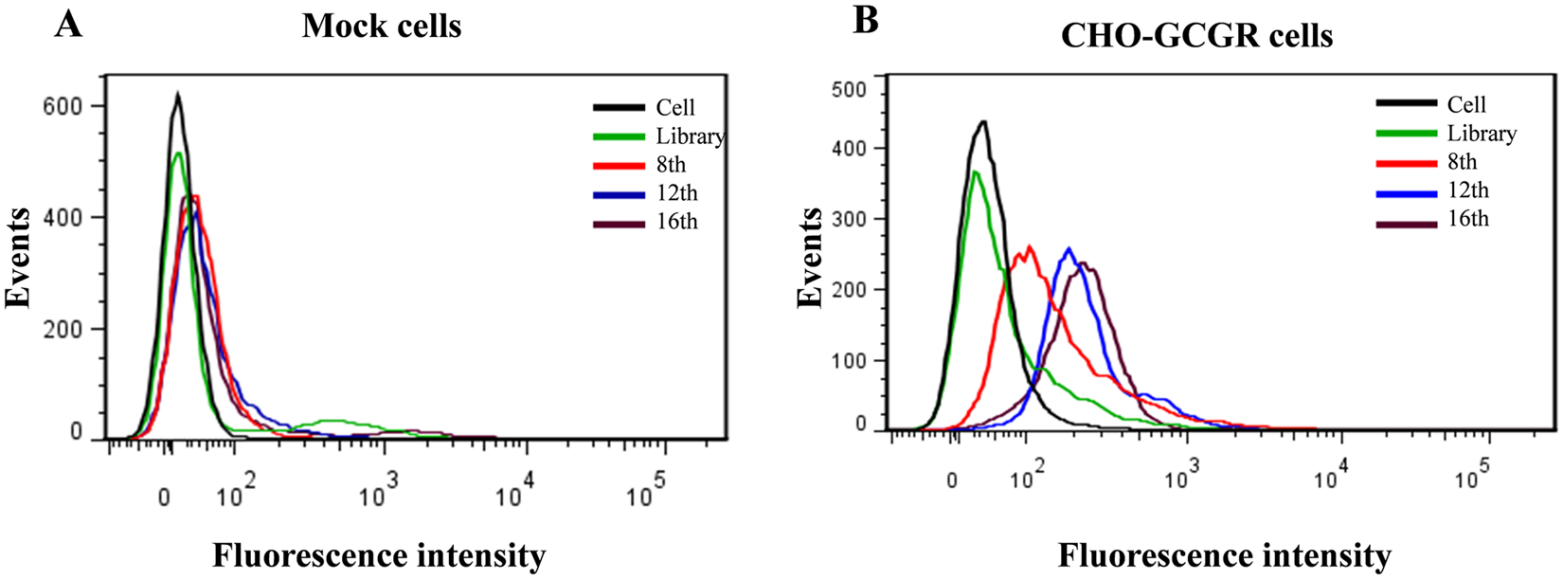
Monitoring the enrichment of cell-SELEX progression. (**A**) Binding of enriched pools to Mock cells (control cells) and (**B**) CHO-GCGR cells (target cells) from the 8th, 12th and 16th rounds were monitored by flow cytometry assay. The unselected initial ssDNA library was used as negative control.

To generate aptamers against GCGR, CHO-K1 cells expressing GCGR (CHO-GCGR) were used for positive selection, and CHO-K1 cells transfected with pcDNA3.1 (Mock cells) were used as negative control. The cell-SELEX method is illustrated in Fig. 1. The enrichment of ssDNA pool was monitored by flow cytometry during selection. Fluorescence intensity reflected the binding capacity of enriched pools. As shown in Fig. 2, the fluorescence intensity on target CHO-GCGR cells was gradually increased after incubation with increasing rounds of ssDNA pools. In contrast, almost no increase in fluorescence signal was observed for negative control Mock cells after incubation with FAM-labeled evolved ssDNA pools. The results suggest that the target cell-binding DNA sequences were gradually enriched during the selection process. The enrichment process finished after the 16th round of selection, and the final ssDNA pool was cloned and high-throughput sequenced with Illumina MiSeq.

### Identification of ssDNA aptamer candidates

**Figure 3:**
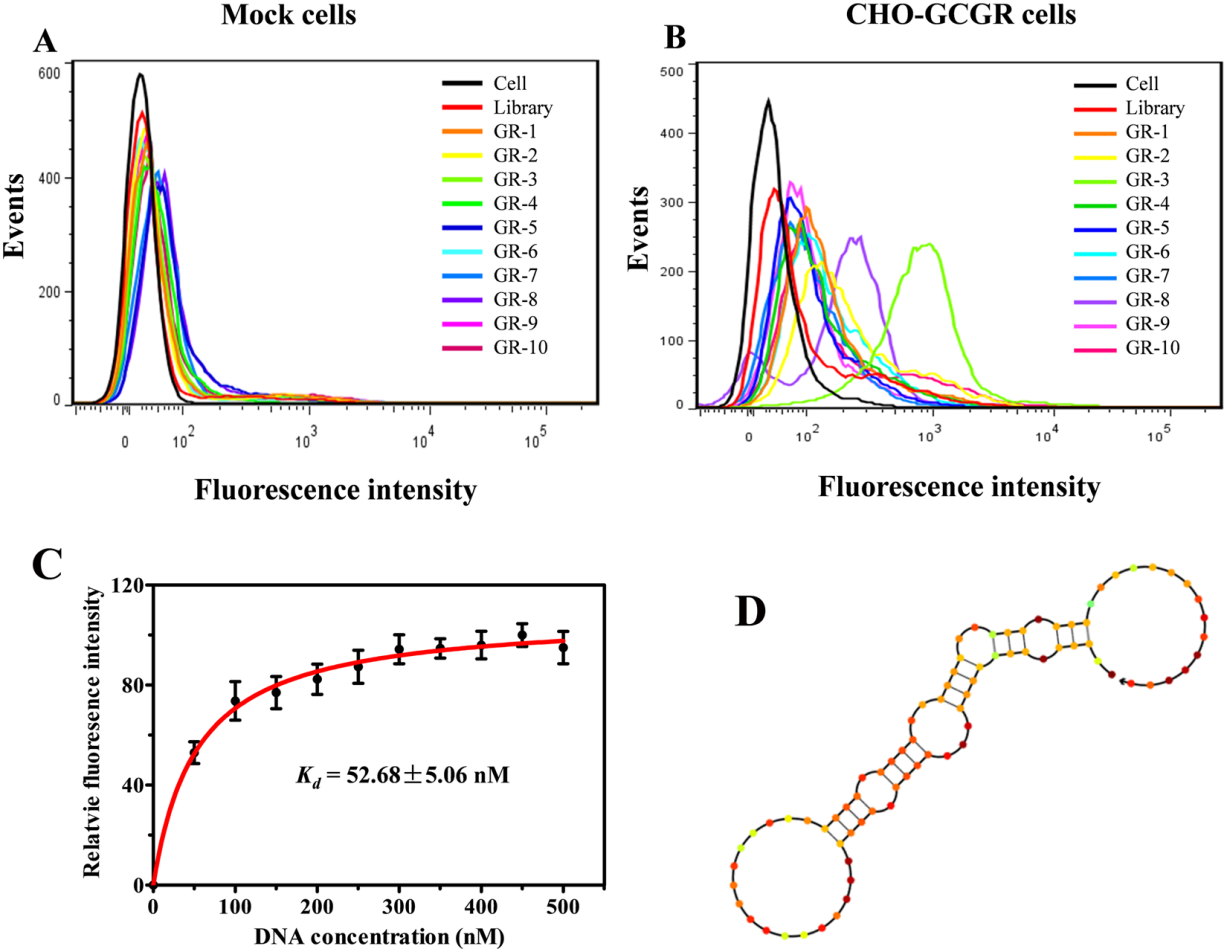
Binding assay of aptamer candidates. The binding ability of aptamer candidates to (**A**) Mock cells and (**B**) CHO-GCGR cells was analyzed by flow cytometry. The black curve represents the background fluorescence of untreated cells. The unselected initial ssDNA library was used as negative control. (**C**) Dissociation constant (*K*_*d*_) of GR-3 for CHO-GCGR cells was measured by flow cytometry. (**D**) Secondary structure of GR-3 predicted by NUPACK.

After sequencing, the aptamer candidates were grouped based on their sequential repeatability, secondary structures and homogeneity. Ten representative sequences from different groups were chosen and chemically synthesized on a DNA synthesizer for further characterization (Supplementary Table S1). Flow cytometry revealed that one of these sequences, termed GR-3, showed the highest selectivity to bind target CHO-GCGR cells, rather than control Mock cells (Fig. 3A,B). These results indicated that aptamer GR-3 might be capable of distinguishing CHO-GCGR cells from Mock cells.

To quantitatively evaluate the binding affinity of GR-3 to CHO-GCGR cells, the equilibrium dissociation constant (*K*_*d*_) was measured. Briefly, CHO-GCGR cells were incubated with different concentrations of FAM-labeled GR-3 or initial library at 4 °C for 45 min, followed by monitoring fluorescence intensity by flow cytometry. The FAM-labeled unselected initial ssDNA library was used as a negative control. After subtracting the geometric mean fluorescence (GMF) intensity of cells incubated with initial library from that of the target cells treated with GR-3, the *K*_*d*_ of GR-3 for CHO-GCGR cells was calculated using the equation *Y* = *B*_*max*_ *X*/(*K*_*d*_ + *X*). As shown in Fig. 3C, aptamer GR-3 was determined to possess a *K*_*d*_ of 52.7 ± 5.1 nM, indicating that the selected aptamer GR-3 could specifically recognize target CHO-GCGR cells with high affinity.

### Truncated aptamer with high affinity to CHO-GCGR cells

The full-length aptamers generated by SELEX contain 81 nucleotides, including two flanked primer sequences on each end for PCR amplification. Generally, not all nucleotides are required for target binding. In fact, longer sequences typically increase production costs, but with lower yield, in DNA synthesis^45^. Thus, many selected aptamers are truncated to a minimal functional sequence after SELEX for further applications^46^. Based on the secondary structure of GR-3 predicted by NUPACK (Fig. 3D), five truncated sequences from GR-3 were synthesized by gradually removing the nucleotides at the 5′ and 3′ termini (Supplementary Table S2). Flow cytometry analysis showed that these truncated sequences could bind to CHO-GCGR cells with different binding affinity, but not to Mock cells (Supplementary Fig. S2). Among these deletion variants, GR-3d maintained high affinity and selectivity against CHO-GCGR cells with minimal influence, thus closely approaching the selectivity of full-length GR-3. These results indicate that neither forward nor reverse primers are necessary for target binding of GR-3.

### Aptamer GR-3 specifically targets GCGR on the surface of CHO-GCGR cells

**Figure 4:**
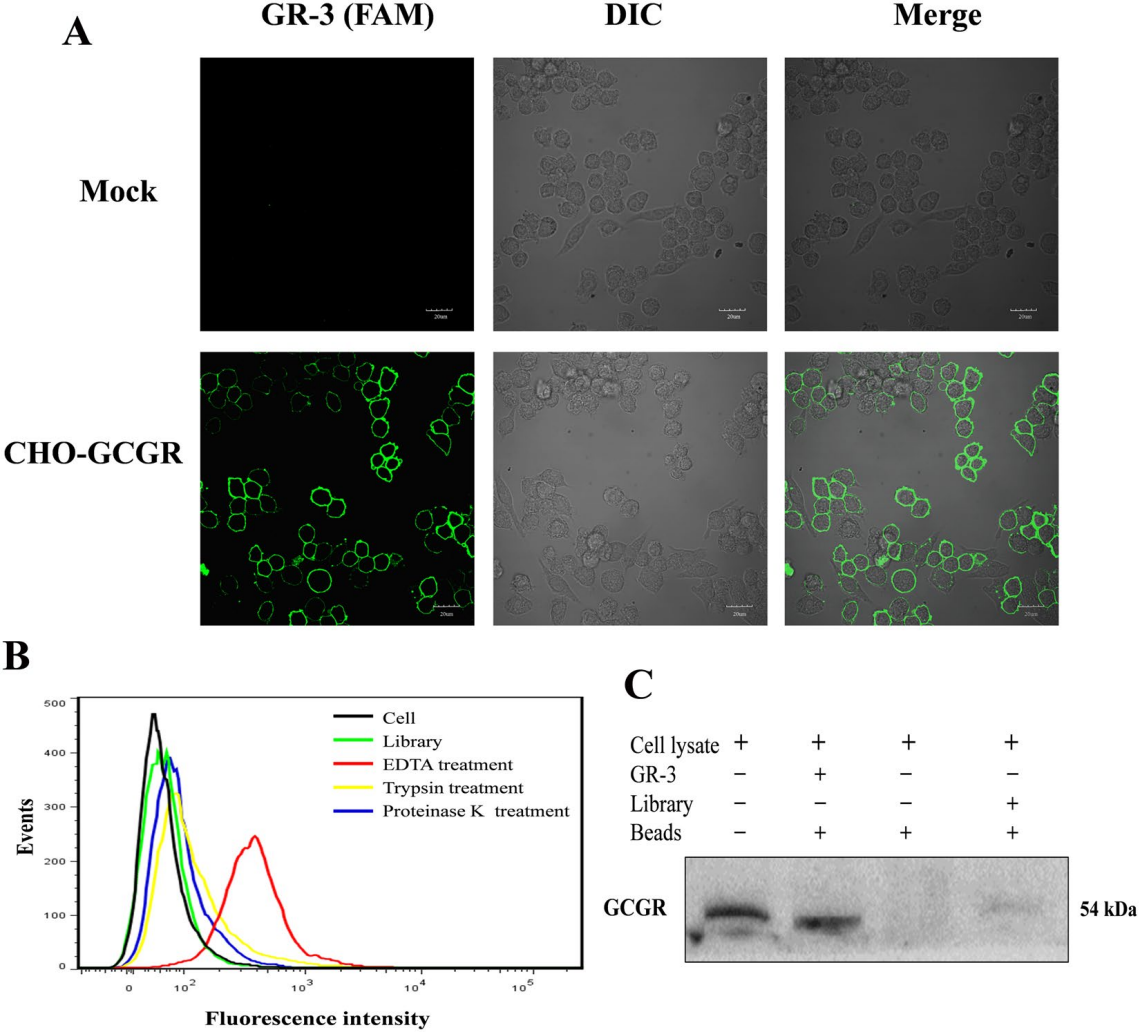
Target location and type analysis of aptamer GR-3. (**A**) Binding site of aptamer GR-3 to CHO-GCGR cells was investigated by confocal microscopy imaging. (**B**) CHO-GCGR cells were treated with trypsin or proteinase K and then incubated with FAM-labeled GR-3 to analyze the target type by flow cytometry. (**C**) Identification of binding affinity between biotin-labeled GR-3 and GCGR protein by aptamer-mediated pull-down assay.

To investigate the binding site of aptamer GR-3 to CHO-GCGR cells, confocal microscopy imaging was performed. The fluorescence signal was observed mainly on the surface of CHO-GCGR cells after incubation with GR-3, but not on Mock cells (Fig. 4A), indicating that the targets of GR-3 were presumably located on the cytomembrane.

In order to elucidate whether the binding target of GR-3 is an extracellular membrane protein, CHO-GCGR cells were treated with trypsin or proteinase K for 10 min, followed by incubating for 1 h with aptamer GR-3. As shown in Fig. 4B, both trypsin and proteinase K treatment completely abolished the binding signal of aptamer GR-3. Proteinases, such as proteinase K and trypsin, could effectively digest extracellular domains of membrane protein without disrupting other components of the plasma membrane, such as lipids, saccharides and other organic molecules^47^. Therefore, these results clearly suggest that the binding targets of GR-3 are most likely membrane proteins.

In order to further determine if aptamer GR-3 targeted the GCGR molecule, we performed an aptamer-mediated pull-down assay based on biotin-streptavidin purification. The cell lysate was incubated with biotin-labeled GR-3, or library, and further enriched by using streptavidin-coated sepharose beads, followed by Western blotting analysis with anti-GCGR antibody. As shown in Fig. 4C, the band of GCGR protein was clearly visualized following aptamer-mediated pull-down assay with biotin-labeled GR-3, similar to cell lysates without any treatment (positive control). In contrast, no band was obtained from the cell lysates incubated with the library or beads alone. These results confirm that GR-3 is able to interact with GCGR from the CHO-GCGR cells and that GCGR is the target molecule of GR-3.

### Effect of incubation temperature on the binding ability of GR-3

**Figure 5:**
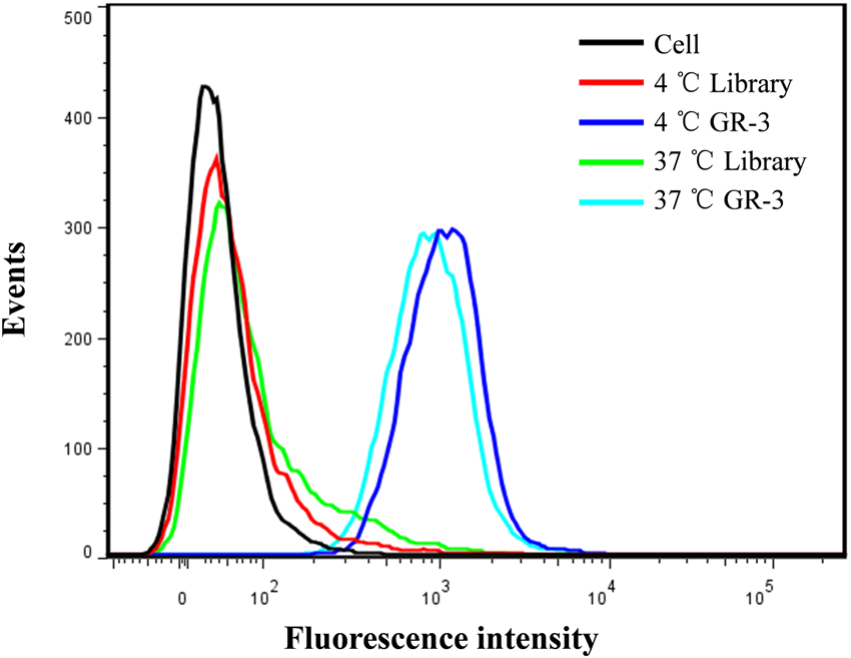
Effect of incubation temperature on binding ability of aptamer GR-3. CHO-GCGR cells were incubated with FAM-labeled GR-3, or library, at 4 °C or 37 °C and then analyzed by flow cytometry.

Cell-SELEX was performed at 4 °C to avoid enriching nonspecific DNA sequences caused by endocytosis at 37 °C. However, selection at 4 °C may lead to aptamers with poor binding capacity at physiological temperature^48, 49^. To investigate whether incubation temperature could affect the binding capacity of GR-3, CHO-GCGR cells were incubated with aptamer GR-3 at different incubation temperatures and further analyzed by flow cytometry. As illustrated in Fig. 5, CHO-GCGR cells presented almost the same fluorescence signals after incubation with aptamer GR-3 at 4 °C or 37 °C, indicating that incubation temperature has little effect on the binding ability of GR-3.

### Binding specificity of GR-3 to other cell lines

**Figure 6:**
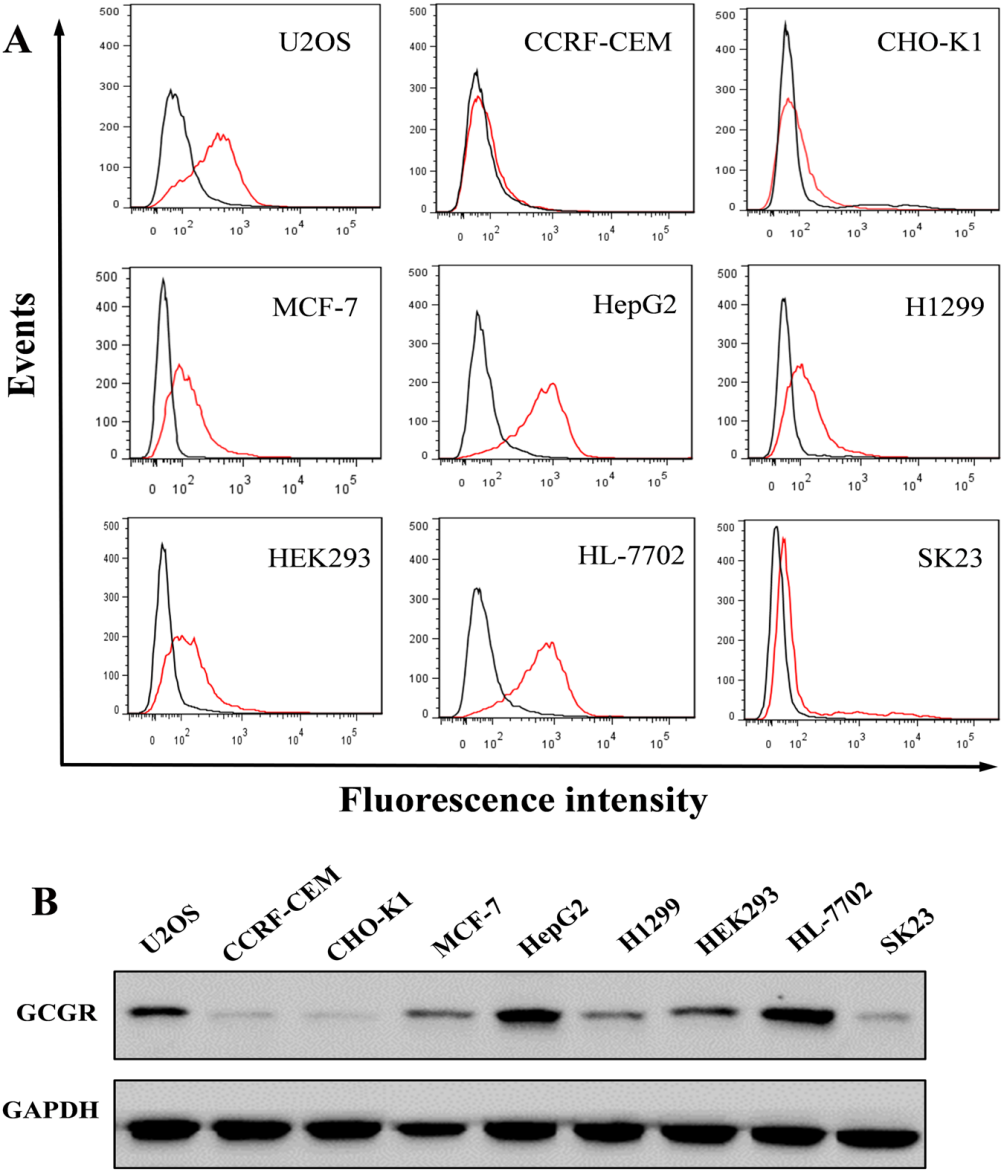
Analysis of binding specificity of aptamer GR-3 to other cell lines. (**A**) FAM-labeled GR-3 (250 nM, red line) was incubated with cell lines of different origins and analyzed by flow cytometry. The unselected initial library (250 nM, black line) was used as control. (**B**) The expression of GCGR protein in different cell lines was analyzed by Western blot. GAPDH was used as an internal control. Original blots are presented in Supplementary Figure S4.

To characterize the binding specificity of aptamer GR-3 to other cell lines, 9 cell lines (U2OS, CCRF-CEM, CHO-K1, MCF-7, HepG2, H1299, HEK293, HL-7702 and SK23) were respectively incubated with 250 nM of FAM-labeled GR-3 for 1 h at 4 °C and evaluated by flow cytometry. The unselected initial library (250 nM) was used as control. In addition, the expression of GCGR in these cells was further analyzed by Western blot with anti-GCGR antibody. As shown in Fig. 6, aptamer GR-3 strongly bound to U2OS, HepG2 and HL-7702 cells, which presented high expression of GCGR in Western blot analysis. On the other hand, GR-3 showed little, or no, binding affinity for CCRF-CEM, CHO-K1 and SK23 cells, which presented low expression of GCGR. Thus, the ability of GR-3 to bind these cells varied in accordance with the protein expression of GCGR. These results indicate that GR-3 could recognize the native GCGR protein in other cells and that the expression of GCGR could affect the binding specificity of GR-3 to other cell lines.

### *Gcgr* Knockdown decreases the binding ability of GR-3

To further elucidate whether the expression of GCGR in cells is essential for the binding ability of GR-3. HepG2 cells with high expression of GCGR were transfected with siRNA targeted against *gcgr* and nonspecific-siRNA. Proteins were extracted from the transfected cells, and the expression of GCGR was detected by Western blotting. Significant decreases of the expression of GCGR was observed in *gcgr* knockdown cells (Supplementary Fig. S3A). Flow cytometry analysis showed that GR-3 scarcely bind to the *gcgr* knockdown cells (Supplementary Fig. S3B). These results firmly confirm that GCGR is the target molecule of aptamer GR-3.

### Imaging of hepatic tissues with aptamer GR-3

**Figure 7:**
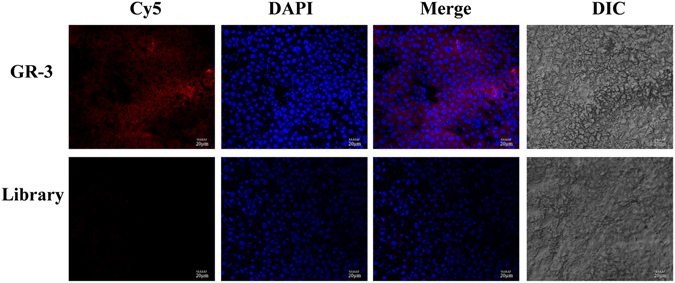
Representative fluorescence images of liver tissue sections stained with Cy5-labled GR-3 or library (250 nM).

Since GCGR is predominantly localized in the liver^3, 4^, we speculate that GR-3 may also have the ability to bind hepatic tissues. To test this hypothesis, laser confocal fluorescence microscopy was used to image frozen sections of mouse hepatic tissue with Cy5-labeled GR-3 (Fig. 7). A red fluorescence signal can be seen on the cell membrane in hepatic tissues after incubation with Cy5-labeled GR-3. In contrast, no staining was obtained using the Cy5-labeled ssDNA library for the analysis of the same tissue sections. These results suggest that GR-3 can bind the cell membrane of hepatic tissues.

## Conclusions

In summary, a DNA aptamer against GCGR has been successfully selected by cell-SELEX after 16 rounds of evolved enrichment. Our selected aptamer, GR-3, can specifically bind to CHO-GCGR cells with *Kd* value in the nanomolar range. The target of aptamer GR-3 was preliminarily verified as a membrane protein on the cell surface. Specifically, aptamer-mediated pull-down and *gcgr* knockdown assay confirmed that GCGR is the target molecule of GR-3. Binding analysis revealed that GR-3 can also recognize other cells with different affinity in accordance with the protein expression level of GCGR in these cells. A truncated sequence GR-3d maintains recognition and binding to CHO-GCGR, similar to that of full-length GR-3. Imaging of hepatic tissue suggests that GR-3 can bind the cell membrane of hepatic tissues. With the advantages of small size, high binding affinity, good stability, lack of immunogenicity, easy synthesis and modification, aptamer GR-3 against GCGR is a promising probe molecule which is a potential tool for the treatment of diabetes mellitus.

## Materials and Methods

All of the experimental protocols for the follow-up study were regulated and approved by Xiangya Hospital, Central South University, and all the methods were performed in accordance with relevant guidelines and regulations.

### Cell lines and cell culture

Chinese hamster ovary cell line CHO-K1, hepatoma cell line HepG2 and breast cancer cell line MCF-7 were purchased from the Type Culture Collection of the Chinese Academy of Sciences (Shanghai, China). Osteosarcoma cell line U2OS, leukemia cell line CCRF-CEM, and Burkitt’s lymphoma Ramos cell line were obtained from the American Type Culture Collection (ATCC). Lung cancer cell line H1299, melanoma cell line (SK23), human embryonic kidney epithelial cell line HEK293 and liver cell line HL-7702 were provided by the cell bank of Xiangya Hospital (Changsha, China). CHO-K1 cells were cultured in Ham’s F-12K medium supplemented with 10% fetal bovine serum (FBS, HyClone). HepG2, MCF-7, HL-7702, SK23 and HEK293 cells were cultured in Dulbecco’s minimal essential medium (DMEM) containing 10% FBS. The growth medium for U2OS, CCRF-CEM, Ramos, and H1299 cells was composed of RPMI-1640 with 10% FBS. All cell lines were cultured at 37 °C in a humid atmosphere with 5% CO_2_.

### Construction of recombinant vector and transfection

Human glucagon receptor (GCGR) cDNA (GeneCopoeia, NM_000160.3) was cloned into a pcDNA3.1 eukaryotic expression vector (Invitrogen), and the resulting expression plasmid pcDNA3.1-GCGR was confirmed by DNA sequencing. CHO-K1 cells were transfected with pcDNA3.1-GCGR plasmid using Lipofectamine 3000 and Opti-MEM serum-free media (Invitrogen) according to the manufacturer’s instructions. The CHO-K1 cell line overexpressing GCGR (CHO-GCGR cells) was treated with Geneticin^®^ reagent for positive selection. PcDNA3.1 plasmid was transfected in CHO-K1 cells (Mock cells) by the same method as that for negative selection. After transfection, the level of GCGR protein was measured by Western blot analysis using primary rabbit polyclonal to GCGR (Abcam). The location of GCGR was determined by confocal microscopy imaging using primary rabbit polyclonal to GCGR and Cy3-conjugated goat polyclonal secondary antibody to rabbit IgG.

### ssDNA library, primers and buffers

HPLC-purified ssDNA library and primers used in cell-SELEX were synthesized by Sangon Biotech Co. Ltd. (Shanghai, China). The library contained a central randomized sequence of 45 nucleotides flanked by 18-nucleotide sequences for primer annealing (5′-ATC CAG AGT GAC GCA GCA (45 N) TGG ACA CGG TGG CTT AGT-3′). The forward and reverse primers, 5′-FAM-ATC CAG AGT GAC GCA GCA-3′ and 5′-Biotin-ACT AAG CCA CCG TGT CCA-3′, respectively, were synthesized for polymerase chain reaction (PCR) amplification. Washing buffer contained 5 mM MgCl_2_ and 4.5 g/L glucose in Dulbecco’s Phosphate Buffered Saline (D-PBS, pH = 7.4). Binding buffer was prepared from washing buffer by adding 0.1 mg/mL yeast tRNA and 1 mg/L bovine serum albumin (BSA) to reduce nonspecific binding.

### Cell-SELEX

Cell-SELEX was performed essentially as described previously with a few modifications^18^. The initial ssDNA library (10 OD) dissolved in 1 mL of binding buffer was denatured at 95 °C for 5 min and then cooled immediately on ice for 10 min to reduce intermolecular hybridization. CHO-GCGR cells in a 100 mm diameter dish were incubated with the initial library for 2 h at 4 °C for positive selection. After incubation, the supernatant was discarded, and cells were washed with 3 mL of washing buffer to remove unbound sequences. The cells were harvested from the culture dish using a cell scraper, and the cell-bound ssDNAs were eluted by heating at 95 °C for 10 min. The supernatant was collected and amplified by PCR using FAM-labeled forward primer and biotin-labeled reverse primer under the following conditions: 95 °C for 3 min, 6–14 cycles of 30 s at 95 °C, 30 s annealing at 55.9 °C, and 30 s extension at 72 °C, followed by 72 °C for 5 min. PCR Taq polymerase and dNTPs were products of Takara. The double-stranded DNA (dsDNA) product in the PCR solution was separated by streptavidin-coated sepharose beads (GE Healthcare). After denaturation in 0.2 M NaOH and desalting with NAP-5 column (GE Healthcare), the FAM-labeled ssDNA pool was lyophilized and used for next round selection or cytometric analysis.

From the third round, negative selection was introduced with Mock cells in a 60 mm diameter dish. Cells were incubated with the selected ssDNA pool at 4 °C for 30 min. The unbound ssDNAs were then removed and subjected to further positive selection. To acquire aptamers with high affinity and specificity, the positive incubation time was decreased from 2 h to 30 min and negative incubation time was gradually increased from 30 min to 2 h to reduce the nonspecific binding of the selected pools. At the same time, washing times were increased from once to 5 times, and the amount of library ssDNA per round was decreased gradually. After 16 rounds of selection, the resulting ssDNA pool was PCR-amplified and sequenced using Illumina MiSeq by Sangon Biotech Co., Ltd. (Shanghai, China).

### Flow cytometric analysis

To investigate the progress of selection, as well as visualize the binding affinity and specificity of aptamer candidates, 250 nM of FAM-labeled ssDNA pools in 200 µL of binding buffer were incubated with 3 × 10^5^ target or negative cells at 4 °C for 1 h. The FAM-labeled initial library was used as a negative control. After incubation, cells were rinsed three times with 500 µL of washing buffer and then resuspended in 500 µL of binding buffer. The fluorescence signal was analyzed with a FACSVerse^TM^ flow cytometer (BD Biosciences, USA).

To measure the dissociation constant (*K*_*d*_) of aptamers, CHO-GCGR cells (3 × 10^5^) were incubated with a series of concentrations of FAM-labeled aptamers in 200 µL of binding buffer at 4 °C for 1 h. The initial library was used as a negative control. Cells were washed three times with washing buffer and resuspended in 500 μL binding buffer for flow cytometric analysis. The *K*_*d*_ of GR-3 was determined by fitting the dependence of fluorescence intensity on aptamer concentration with the equation *Y* = *B*_*max*_ *X*/(*K*_*d*_ + *X*). All of the experiments for the binding assay were repeated in triplicate. To investigate the selectivity of aptamers for molecular recognition, the other cell lines described above were applied for binding specificity assays with FAM-labeled aptamer candidates by flow cytometry.

### Confocal microscopy imaging

Confocal microscopy imaging was performed to monitor the binding of aptamer to live cells. Cells (1 × 10^5^) were seeded in a 35 mm glass bottom dish and cultured overnight. After washing twice with cold washing buffer, the cells were incubated with FAM- or Cy5-labeled aptamers (250 nM) in binding buffer at 4 °C for 1 h. Subsequently, the cells were washed twice and imaged using a confocal laser scanning microscope (CLSM, Olympus, Japan).

### Effects of proteinase treatment and temperature on aptamer binding

For target type analysis, 70–80% confluent CHO-GCGR cells were washed twice with D-PBS and dissociated with 200 μL 0.25% trypsin, 0.1 mg/ml Proteinase K or 0.02% EDTA at room temperature for 10 min, respectively. After adding FBS to inhibit proteinase activity and washing twice with washing buffer, cells were incubated with 250 nM of FAM-labeled aptamer in 200 µL of binding buffer at 4 °C for 1 h and further analyzed by flow cytometry.

To investigate the effect of temperature on aptamer binding, CHO-GCGR cells were detached with 0.02% EDTA and incubated with 250 nM of FAM-labeled aptamer in 200 µL of binding buffer at 4 °C or 37 °C for 1 h, respectively. After washing with washing buffer, cell samples were analyzed by flow cytometry.

### Aptamer-mediated pull-down assay

The membrane proteins were prepared as described previously with a few modifications^50–52^. CHO-GCGR cells were suspended in hypotonic buffer [50 mM Tris-HCl, 0.1 mM PMSF and a protease inhibitor cocktail] at 4 °C for 30 min. After centrifugation at 1,000 *g*, the debris was washed three times with hypotonic buffer and dissolved in lysis buffer (hypotonic buffer containing 5 mM MgCl_2_ and 2% Triton X-100) at 4 °C for 30 min. After centrifugation at 5,500 *g* and 4 °C, the supernatant was collected and the protein concentration was quantified using the BCA Protein Assay Reagent kit (Beyotime, Jiangsu, China). One hundred μg of protein per sample was blocked with binding buffer supplemented with 20% FBS and 0.1 mg/ml Salmon Sperm DNA for 60 min, and then incubated with 150 pmol biotin-labeled aptamer GR-3 and library, respectively, at 4 °C overnight. Subsequently, the protein-aptamer complexes were incubated with 20 μL streptavidin-coated sepharose beads (GE Healthcare, Uppsala, Sweden) for another 1 h at 4 °C with rotation. The negative control was incubated with beads alone, while positive control consisted of cell lysates with no treatment. After incubation, the beads were washed three times with D-PBS, and the target-bound proteins were finally eluted by heating at 95 °C for 10 min in 30 μL of 2 × sodium dodecyl sulfate polyacrylamide gel electrophoresis (SDS-PAGE) sample buffer, which was then analyzed by Western blot with anti-GCGR antibody (Abcam, Cambridge, UK).

### Western blot analysis

Western blot was performed as described in our previous study^53^. Samples were loaded into a 10% (w/v) SDS-PAGE gel and transferred onto a polyvinylidene difluoride (PVDF) membrane (Millipore, USA). After blocking in blocking buffer (150 mM NaCl, 20 mM Tris–HCl, 0.1% Tween 20, and 5% nonfat milk) for 1 h at room temperature, the membrane was incubated with the primary antibody against GCGR overnight at 4 °C. After washing 3 times with TBST (150 mM NaCl, 20 mM Tris–HCl, and 0.1% Tween 20), the immunoblot was incubated with a horseradish-conjugated goat anti-rabbit IgG secondary antibody (Santa Cruz, CA, USA) for 1 h. The immunoreactive bands were detected by an enhanced chemiluminescence (ECL) kit (Beyotime, Jiangsu, China).

### Small Interfering RNA interference

The siRNAs targeted against *gcgr* were obtained from RiboBio (Guangzhou, China) and the sequences of siRNAs are listed in Supplementary Table S3. A nonspecific-siRNA was used as negative control (NC). HepG2 cells were transfected with 20 nM siRNA using Lipofectamine RNAiMAX (Invitrogen, Carlsbad, CA) according to the manufacturer’s protocol. Knockdown efficiency was measured by Western blot. The binding ability of GR-3 to HepG2 cells transfected with siRNAs was assessed by flow cytometric analysis.

### Staining of hepatic tissue frozen sections using selected aptamer

Hepatic tissue samples were previously collected from C57BL/6 mice (SLAC Laboratory Animal Co., Ltd, Shanghai, China), as previously approved by the Animal Care and Use Committee of Xiangya Hospital, Central South University^54^. These tissues were embedded in optimal cutting medium (OCT) and stored at −80 °C. The frozen tissue sections were warmed to −20 °C, cut into 8 μm sections using a Leica CM 1950 cryostat (Leica, Wetzlar, Germany), and then mounted on charged glass slides. The prepared tissue sections were blocked with binding buffer supplemented with 20% FBS and 0.1 mg/ml Salmon Sperm DNA for 60 min and then incubated with 200 µL of 250 nM Cy5-labeled aptamer or library in binding buffer on ice in the dark. After incubation for 1 h these tissue sections were washed three times with washing buffer and counterstained with DAPI. The fluorescence signals were then detected using FV10-ASW, v.3.1, software (Olympus).

## Electronic supplementary material

Supplementary Information
