# Supplementary material for: Selection and characterization of DNA aptamer against glucagon receptor by cell-SELEX
Source: Sci Rep. 2017 Aug 3;7:7179. doi: 10.1038/s41598-017-05840-w (PMC5543139; doi:10.1038/s41598-017-05840-w)
Supplement: Supplementary file 1 — Supplementary Information [file 41598_2017_5840_MOESM1_ESM.pdf]

Supplementary information

Selection and characterization of DNA aptamer against glucagon receptor by cell-SELEX

Guodong Wang†, Jun Liu†, Ke Chen, Yiling Xu, Bo Liu, Jie Liao, Lei Zhu, Xiaoxiao Hu, Jianglin Li, Ying Pu, Wen Zhong, Ting Fu, Huixia Liu*, Weihong Tan*

†Both authors contributed equally to this work.

*Correspondence and requests for materials should be addressed to H.L. (email: lhx900@aliyun.com) or W.T. (email: tan@chem.ufl.edu)

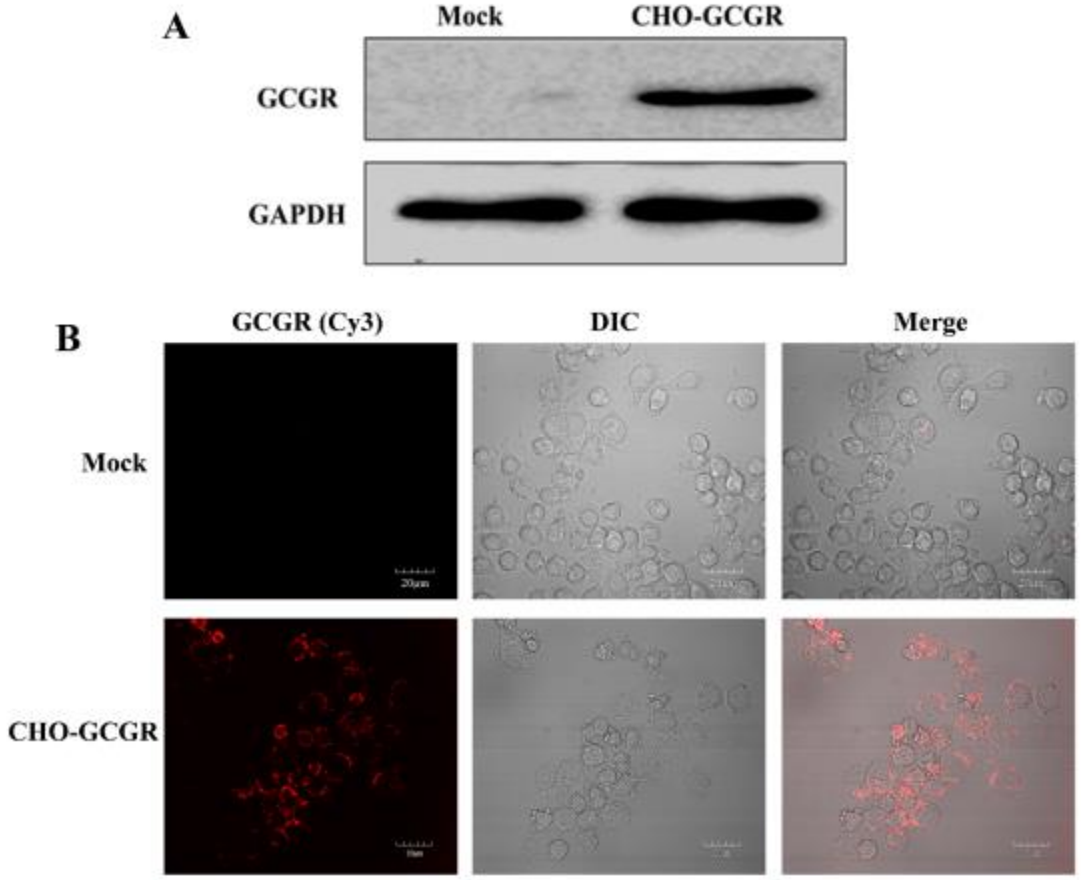

Supplementary Figure S1. The expression level and location of GCGR in CHO-GCGR cells respectively detected by Western blot analysis (A) and confocal microscopic analysis (B).

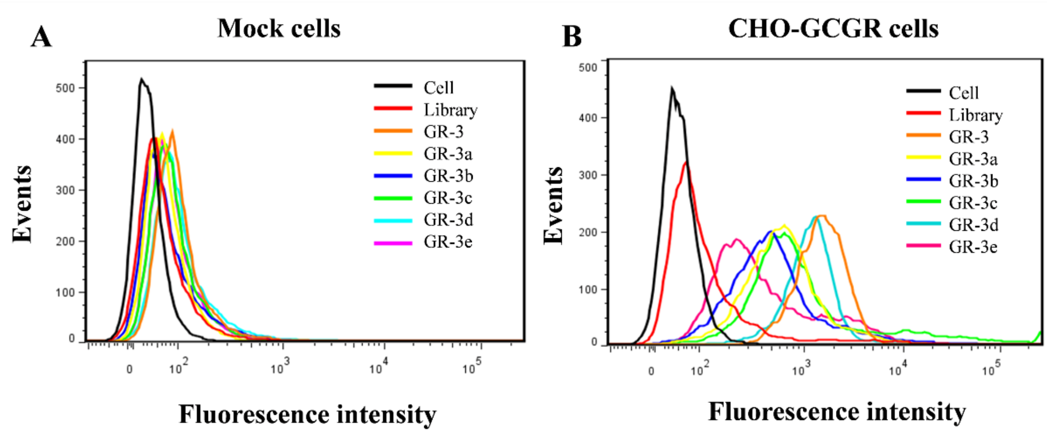

Supplementary Figure S2. Binding ability of different truncated sequences of GR-3 on Mock cells (A) and CHO-GCGR cells (B) assayed by flow cytometry.

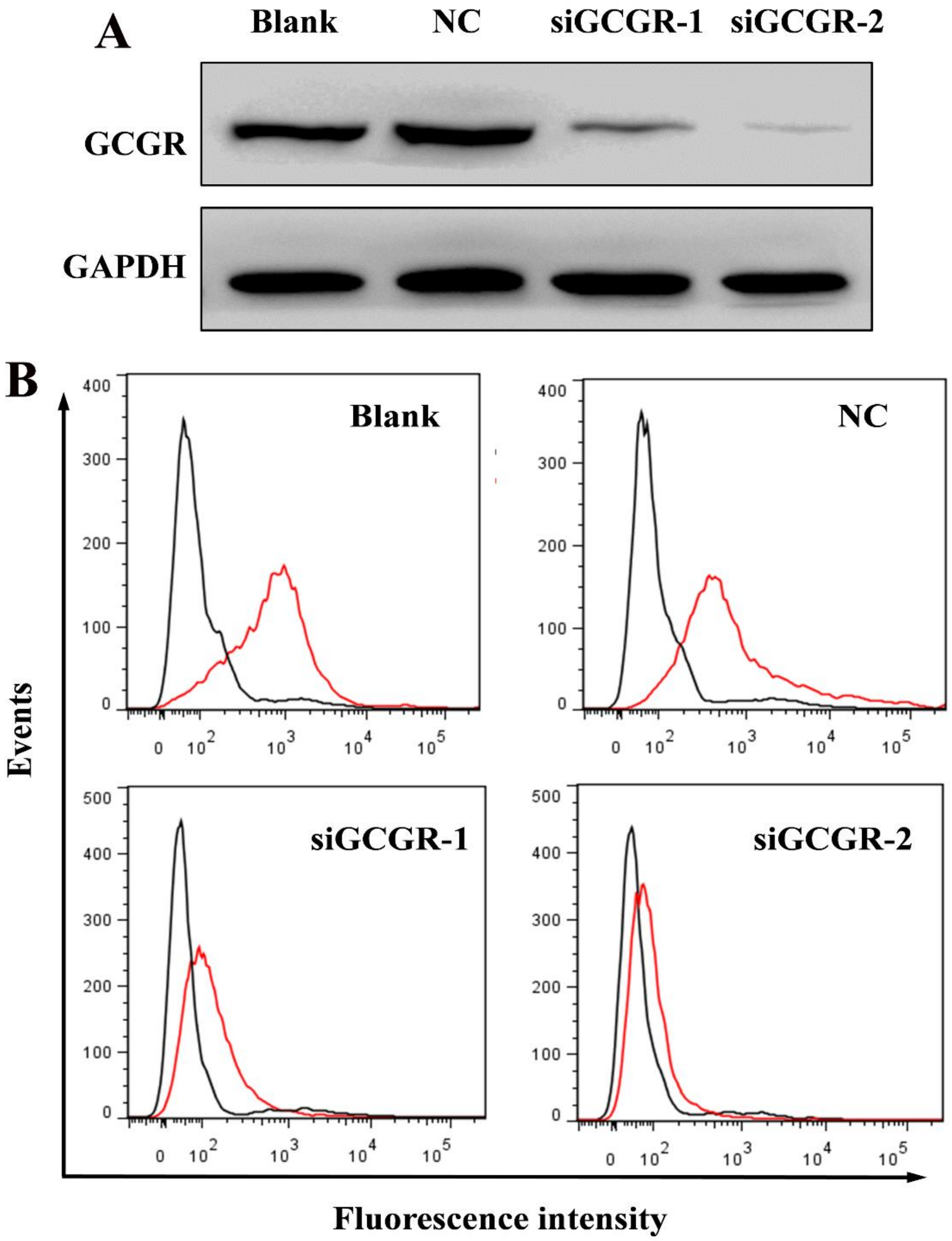

Supplementary Figure S3. Analysis of binding ability of aptamer GR-3 to HepG2 cells transfected with siRNAs. (**A**) Western blot analysis of the expression of GCGR in native HepG2 cells (Blank) and HepG2 cells transfected with nonspecific-siRNA (NC) or siRNA targeted against *gcgr* (siGCGR-1 and siGCGR-2). (**B**) Flow cytometry analysis of the binding of aptamer GR-3 (250 nM, red line) to HepG2 cells transfected with siRNAs. The unselected initial library (250 nM, black line) was used as control.

Supplementary Figure S4. Original blots.

Figure 4C

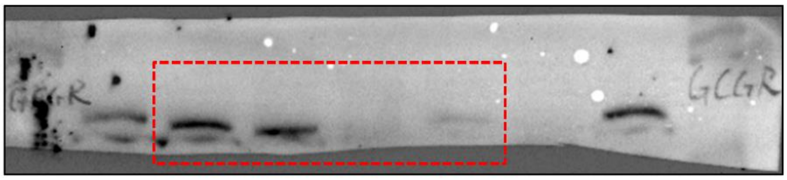

GCGR

Figure 6B

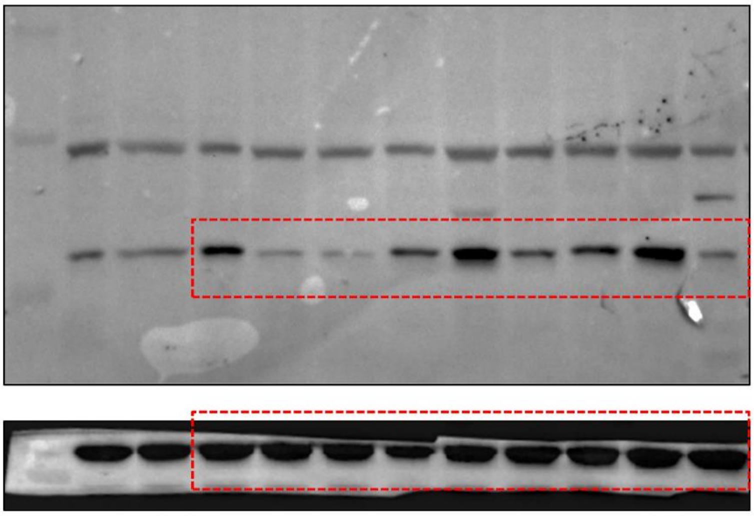

GCGR

GAPDH

Supplementary Table S1. Sequences of aptamer candidates to CHO-GCGR cells

Name	Sequences
GR-1	ATCCAGAGTGACGCAGCAGCCAAAATACTTCTTGTAATTAGGAAG AAAATTCACGCATTTTCTGGACACGGTGGCTTAGT
GR-2	ATCCAGAGTGACGCAGCACGAACATCTAAGGCAATAAATGCCTCT GGTTATTTATAGGAAGAATGGACACGGTGGCTTAGT
GR-3	ATCCAGAGTGACGCAGCAGATAAGTAGGTATCCGTTTGAAAAACT TTTCTGACCGTCCGACTATGGACACGGTGGCTTAGT
GR-4	ATCCAGAGTGACGCAGCACGAATATCTAGGACAATAAATGCCTCT GGTTATTTATAGGAAGAATGGACACGGTGGCTTAGT
GR-5	ATCCAGAGTGACGCAGCACGAATATCTAAGGCAATAAATGCCTCT GGTTATTTATAGGAAGAATGGACACGGTGGCTTAGT
GR-6	ATCCAGAGTGACGCAGCACGACTAAGACAAAAATCTTTACGATA GTTAGGAACCCTTCAGAGGTGGACACGGTGGCTTAGT
GR-7	ATCCAGAGTGACGCAGCACAAAGACAAATTACCTTCGGAGGTAA TTAGGAAAACCTTACAGTTTGGACACGGTGGCTTAGT
GR-8	ATCCAGAGTGACGCAGCACAAAGACAAAATACCTTCGGAGGTAA TTAGGAAAACCCTACAGTTTGGACACGGTGGCTTAGT
GR-9	ATCCAGAGTGACGCAGCAGATAAGTAGGTATCCGTCTGAAAGAC TTTTCTGACCGTCCGACTATGGACACGGTGGCTTAGT
GR-10	ATCCAGAGTGACGCAGCAGATAAGTAGGTATCCGTCTGAAAGAC TTATTTGACCGTCCGACTATGGACACGGTGGCTTAGT

Supplementary Table S2. Truncated sequences from GR-3

Name	Sequences
GR-3a	ATCCAGAGTGACGCAGCAGATAAGTAGGTATCCGTTTGAAAAACTTTTCTGACCGTCCGACTATGGA
GR-3b	GCAGCAGATAAGTAGGTATCCGTTTGAAAAACTTTTCTGACCGTCCGACTATGGACACGGTGGCTTAGT
GR-3c	GACGCAGCAGATAAGTAGGTATCCGTTTGAAAAACTTTTCTGACCGTCCGACTATGGAC
GR-3d	GCAGCAGATAAGTAGGTATCCGTTTGAAAAACTTTTCTGACCGTCCGACTATGGA
GR-3e	ATAAGTAGGTATCCGTTTGAAAAACTTTTCTGACCGTCCGACTATGGA

Supplementary Table S3. The sequences of siRNAs

siRNA	Sense(5'→3')	Antisense(5'→3')
si-GCGR-1	UGUGCAACAGAACCUUCGA(dTdT)	UCGAAGGUUCUGUUGCACA(dTdT)
si-GCGR-2	CAAUGCCAUCCACGCGAAU(dTdT)	AUUCGCGUGGAUGGCAUUG(dTdT)
negative control (NC)	UUCUCCGAACGUGUCACGU(dTdT)	ACGUGACACGUUCGGAGAA(dTdT)
